# Yokukansan and Yokukansankachimpihange Ameliorate Aggressive Behaviors in Rats with Cholinergic Degeneration in the Nucleus Basalis of Meynert

**DOI:** 10.3389/fphar.2017.00235

**Published:** 2017-04-26

**Authors:** Masahiro Tabuchi, Keita Mizuno, Kazushige Mizoguchi, Tomohisa Hattori, Yoshio Kase

**Affiliations:** Tsumura Research Laboratories, Kampo Scientific Strategies Division, Tsumura & Co.Ami-machi, Japan

**Keywords:** aggressive behavior, glutamic acid, nucleus basalis of Meynert, rats, yokukansan, yokukansankachimpihange

## Abstract

Yokukansan (YKS) and yokukansankachimpihange (YKSCH) are traditional Japanese Kampo medicines. The latter comprises YKS along with the medicinal herbs *Citrus unshiu* peel and *Pinellia* tuber. Both of these Kampo medicines are indicated for the treatment of night crying and irritability in children and for neurosis and insomnia in adults. In recent clinical trials, YKS exhibited ameliorative effects on the behavioral and psychological symptoms of dementia, such as aggressiveness, excitement, and irritability. In the present study, we aimed to clarify the involvement of cholinergic degeneration in the nucleus basalis of Meynert (NBM) in the development of aggressiveness in rats. Subsequently, using this animal model, the effects of YKS and YKSCH on aggressiveness were compared and the mechanisms underlying these effects were investigated. L-Glutamic acid (Glu) was injected into the right NBM of rats to induce deterioration of cholinergic neurons. On day 8 after Glu injection, aggressive behaviors were evaluated using resident–intruder tests. After the evaluation, YKS or YKSCH was administered to rats with aggressive behaviors daily for 7 days. In some groups, the 5-HT_1A_ receptor antagonist WAY-100635 was coadministered with YKS or YKSCH over the same period. In other groups, locomotor activity was measured on days 12–14 after Glu injection. On day 15, immunohistochemistry was then performed to examine choline acetyltransferase (ChAT) activities in the NBM. Aggressive behaviors had developed on day 8 after Glu injection and were maintained until day 15. YKS and YKSCH significantly ameliorated the aggressive behaviors. These suppressive effects were entirely abolished following coadministration of WAY-100635. Finally, the number of ChAT-positive cells in the right NBM was significantly reduced on day 15 after Glu injection, and treatment with YKS or YKSCH did not ameliorate these reduced cell numbers. Our results show that unilateral Glu injections into the NBM of rats leads to the development of aggressive behaviors, which is thought to reflect cholinergic degeneration. YKS and YKSCH treatments ameliorated Glu-induced aggressive behaviors, and these effects were suggested to be mediated by 5-HT_1A_ receptor stimulation, but not by improvement of cholinergic degeneration.

## Introduction

Degeneration of cholinergic neurons in the nucleus basalis of Meynert (NBM) is believed to contribute to the development of various progressive neurodegenerative diseases, including Alzheimer’s disease (AD), dementia with Lewy bodies, Parkinson’s disease, Korsakoff’s syndrome, and Down’s syndrome (Arendt et al., 1995; Bohnen and Albin, 2011). This degeneration is generally thought to cause cognitive deficits and emotional disturbances in dementia patients (Arendt et al., 1995; Bohnen and Albin, 2011; Mesulam, 2013). Activation of cholinergic neurons using cholinesterase inhibitors, such as donepezil, rivastigmine, and galantamine, is a treatment strategy for the cognitive deficits and behavioral and psychological symptoms of dementia (BPSD), such as aggressiveness, excitement, and anxiety (Rodda et al., 2009). These findings suggest that cholinergic degeneration in the NBM is critically involved in the pathogenesis of dementia.

Although the causes of cholinergic degeneration remain unknown, excitotoxicity due to excessive release of endogenous glutamate is considered to be a pathogenic contributor to neuronal death (Maragas et al., 1987; Francis, 2003; Montiel et al., 2005). Excessive glutamate release is also associated with cognitive dysfunction and agitation/aggression in AD patients (Reisberg et al., 2003; Gauthier et al., 2008). In animals, learning and memory impairments (Dunnett et al., 1987; Markowska et al., 1990; Biggan et al., 1991; Boegman et al., 1992; Harkany et al., 1999) and BPSD-like symptoms including anxiety and hypoactivity (Harkany et al., 1999, 2000, 2001; Burk and Sarter, 2001) have been observed in rats following unilateral or bilateral injection of glutamate receptor agonists, β-amyloid protein, or cholinergic neurotoxin into the NBM. Although these findings suggest that cholinergic degeneration in the NBM is induced by glutamate excitotoxicity during the development of cognitive deficits, anxiety, and hypoactivity, the causes of aggressiveness remain unclear.

Yokukansan (YKS) and yokukansankachimpihange (YKSCH) are traditional Japanese “Kampo” medicines. The latter comprises YKS with the two medicinal herbs *Citrus unshiu* peel and *Pinellia* tuber (*Pinellia ternata*), and both medicines have been approved by the Japanese Ministry of Health, Labour, and Welfare for the treatment of neurosis and insomnia, as well as night crying and peevishness in children. In clinical trials, YKS was found to be an effective treatment for positive BPSD, such as aggressiveness, excitement, irritability, and hallucinations (Iwasaki et al., 2005; Mizukami et al., 2009). Moreover, in animals, YKS ameliorated aggressive behaviors induced by thiamine deficiency (Ikarashi et al., 2009; Iizuka et al., 2010), zinc deficiency (Takeda et al., 2008, 2012), isolation stress (Nishi et al., 2012), Aβ deposition in the brain (Fujiwara et al., 2011), and intracerebroventricular injection of Aβ oligomers (Sekiguchi et al., 2011). These effects were considered reflective of glutamatergic and serotonergic regulatory changes. Regarding the glutamatergic system, YKS attenuated excessive glutamate release (Takeda et al., 2008) and improved reduced glutamate transporter function and glutamate-induced neuronal death (Kawakami et al., 2009, 2010). In the serotonergic system, YKS exhibited partial agonistic actions for serotonin 1A (5-HT_1A_) receptors and upregulated the receptors (Terawaki et al., 2010; Nishi et al., 2012; Ueki et al., 2015). However, the effects of YKS on aggressiveness due to cholinergic neuronal degeneration in the NBM, which mostly reflects the pathogenesis of AD, have not been investigated.

Although YKSCH is indicated for the same conditions as YKS, pharmacological evidence of its efficacy against BPSD-like symptoms is lacking. Moreover, whether YKSCH has equivalent pharmacological potency to YKS remains unclear. Interestingly, the traditional uses of YKSCH differ somewhat from those of YKS, that is, YKSCH is recommended for the treatment of patients with more severe losses of physical strength and more chronic conditions.

The first aim of the present study was to elucidate the relationships between cholinergic degeneration in the NBM due to glutamate excitotoxicity and the development of aggressiveness. For this purpose, glutamate was injected into the NBM of rats and the resulting aggressive behaviors were monitored. Subsequently, we evaluated the effects of YKS and YKSCH on glutamate-induced aggressive behaviors and compared their effects. Finally, the mechanisms underlying the ameliorative effects of these drugs on aggressive behaviors were examined in terms of 5-HT_1A_ receptor functions and cholinergic degeneration.

## Materials and Methods

### General Procedures

**FIGURE 1:**
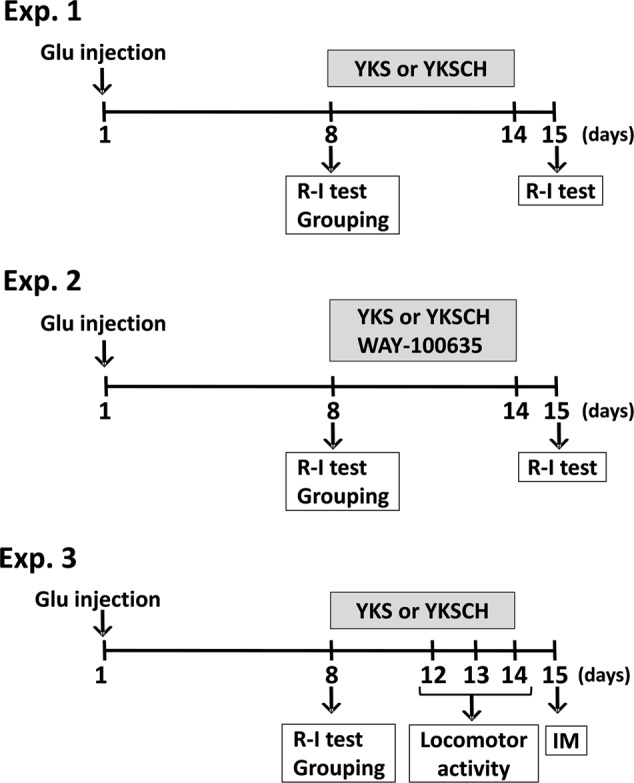
**Schedule of experiments.** In the present study, three experiments were performed. Experiment 1 (Exp. 1): Examination of the development of aggressive behaviors following Glu injection and evaluation of the effects of YKS and YKSCH on the aggressive behaviors. Glu (40 nmol in 1 μL/head) or S-PB (1 μL/head) was injected into the right side of the NBM. On day 8 after injection, aggressive behaviors were analyzed using the R–I test. Glu-injected rats with aggressive behaviors were divided into groups, such that the total number of aggressive behaviors exhibited by the rats during the R–I test was the same for each group. YKS or YKSCH was administered daily for 7 days. On day 15 after injection, aggressive behaviors were analyzed using the R–I test. Experiment 2 (Exp. 2): Evaluation of the effect of 5-HT_1A_ receptor antagonist WAY-100635 upon coadministration with YKS or YKSCH. Glu-injected rats with aggressive behaviors were administered with YKS or YKSCH in the presence of WAY-100635. Experiment 3 (Exp. 3): Measurement of locomotor activity and immunohistochemical detection of cholinergic neurons in the NBM area of Glu-injected rats administered YKS or YKSCH. Locomotor activity was measured on days 12–14 after Glu injection. On the day after final drug treatment (day 15), immunohistochemical staining analyses of the cholinergic neurons in the NBM were performed. Glu, L-glutamic acid; NBM, nucleus basalis of Meynert; R–I test, resident–intruder test; YKS, yokukansan; YKSCH, yokukansankachimpihange; IM, immunohistochemistry.

**Figure 1** shows the schedule comprised of three experiments. In Experiment 1, L-glutamic acid (Glu; 40 nmol in 1 μL/head) or 1/15 M Sørensen’s phosphate buffer containing 41 mM NaCl, pH 7.4 (S-PB: 1 μL/head) was injected into the right side of the NBM. On day 8 after injection, the rats were examined for the development of aggressive behaviors using the resident–intruder (R–I) test. Then, the Glu-injected rats exhibiting aggressive behaviors were divided into groups, such that the total number of aggressive behaviors exhibited by the rats during the R–I test was the same for each group. YKS or YKSCH was administered daily for 7 days. On day 15 after injection, the aggressive behaviors of the rats were analyzed using the R–I test to evaluate the effects of the drugs. In Experiment 2, Glu-injected rats with aggressive behaviors were administered daily with YKS or YKSCH in the presence of the 5-HT_1A_ receptor antagonist WAY-100635 for 7 days. On day 15 after injection, the aggressive behaviors of the rats were analyzed to evaluate the effect of WAY-100635 on the ameliorative actions of YKS or YKSCH. In Experiment 3, measurements of locomotor activity and immunohistochemical detection of cholinergic neurons in the NBM area were performed for the Glu-injected rats administered with YKS or YKSCH. Locomotor activity was measured on days 12–14 after the Glu injections. On the day after the final drug treatments (day 15), immunohistochemical staining analyses of the cholinergic neurons in the NBM of the rats were performed.

### Animals

Six-week-old male Wistar rats were obtained from Charles River Laboratories (Yokohama, Japan). After habituation for 1 week, animals were housed individually in plastic cages (410 mm × 270 mm × 200 mm; Ishihara Co., Ltd., Tokyo, Japan) for the experimental period. Confronted rats were housed in groups of three rats per cage for R–I tests. All animals were maintained at 23 ± 3°C with a relative humidity of 50 ± 20% and a 12-h light/12-h dark cycle with lights on from 07:00 to 19:00, and were allowed free access to water and standard laboratory food (MF; Oriental Yeast Co., Ltd., Tokyo, Japan).

This study was performed in accordance with the recommendations of the Guidelines for the Care and Use of Laboratory Animals of the Japanese Association for Laboratory Animal Science. The protocol was approved by the Ethics Committees for Animal Experiments of Tsumura & Co.

### Reagents and Drugs

L-Glutamic acid was purchased from Wako Pure Chemical Industries (Osaka, Japan), and the selective 5-HT_1A_ receptor antagonist *N*-{2-[4-(2-methoxyphenyl)-1-piperazinyl]ethyl}-*N*-(2-pyridinyl)cyclohexanecarboxamide trihydrochloride (WAY-100635) maleate salt was purchased from Sigma–Aldrich (St. Louis MO, USA). All other chemicals were purchased from commercial sources.

Dry powdered extracts of YKS and YKSCH were supplied by Tsumura & Co. (Tokyo, Japan). YKS comprises seven dried medicinal herbs, including *Atractylodes lancea* rhizome (4.0 g, rhizome of *Atractylodes lancea* De Candolle), *Poria* sclerotium (4.0 g, sclerotium of *Wolfiporia cocos* Ryvarden et Gilbertson), *Cnidium* rhizome (3.0 g, rhizome of *Cnidium officinale* Makino), *Uncaria* hook (3.0 g, hook of *Uncaria rhynchophylla* Miquel), Japanese *Angelica* root (3.0 g, root of *Angelica acutiloba* Kitagawa), *Bupleurum* root (2.0 g, root of *Bupleurum falcatum* Linne), and *Glycyrrhiza* (1.5 g, root and stolon of *Glycyrrhiza uralensis* Fisher). YKSCH comprises YKS with the two additional herbs *Pinellia* tuber (5.0 g, tuber of *Pinellia ternata* Breitenbach, hange) and *Citrus unshiu* peel (3.0 g, peel of *Citrus unshiu* Markovich, chimpi). Plant materials were authenticated by identification of external morphology and marker compounds for plant specimens according to the methods of the Japanese Pharmacopeia and company standards. The seven or nine material herbs were extracted with purified water at 95°C for 1 h, and the extracts were then separated from insoluble waste and concentrated under reduced pressure. Dried extract powders were then produced using spray drying. Extract qualities were standardized based on the good manufacturing practice as defined by the Ministry of Health, Labour, and Welfare of Japan. The yields of YKS and YKSCH were 15.9 and 15.8%, respectively.

Yokukansan (1.0 g) and YKSCH extracts (1.4 g) were dissolved in 10 mL of distilled water (DW). The dosage of YKS (1.0 g/10 mL/kg) was selected according to previous studies, in which treatments with 1.0 g/10 mL/kg of YKS significantly ameliorated aggressiveness, impaired sociality, and anxiety in animal models of neuropsychiatric disorders (Kanno et al., 2009; Sekiguchi et al., 2011; Nishi et al., 2012). YKSCH doses (1.4 g/10 mL/kg) contained the same amounts of the YKS constituents (1.0 g/10 mL/kg).

### Intracranial Injections of Glu

Seven-week-old rats were anesthetized with intraperitoneal (i.p.) injections of sodium pentobarbital (50 mg/mL/kg) and fixed on a stereotaxic apparatus. The rats were then administered a single intracranial injection of 40 nmol Glu in 1.0 μL of S-PB into the right NBM (posterior, 1.5 mm; right lateral, 3.2 mm from the bregma; and ventral, 6.3 mm from the dura), according to a rat brain atlas (Paxinos and Watson, 2007). Control rats received 1.0 μL intracranial injections of S-PB into the right NBM. Injections were performed using a Hamilton Microsyringe at an injection rate of 0.1 μL/min.

### Resident–Intruder (R–I) Tests

Aggressive behavior was evaluated using R–I tests as described previously (Moechars et al., 1998; Takeda et al., 2012). Briefly, a naive untreated age-matched group-housed rat (intruder) was placed in the cage in which a subject rat (resident) had been bred, and interactive behaviors were observed for 10 min. The total number of aggressive behaviors (aggressive grooming, biting attacks, and wrestling) by the resident rats was recorded as an index of aggressiveness.

### Drug Treatments

#### Administration of YKS and YKSCH

Rats (*n* = 26) were divided into vehicle-injected (control group, *n* = 8) and Glu-injected (Glu group, *n* = 18) groups. Glu was then injected into the right NBM of rats in the Glu group (day 1), and aggressive behavior was evaluated using R–I tests on day 8 after the injections. Rats were further divided into Glu + DW (*n* = 6), Glu + YKS (*n* = 6), and Glu + YKSCH (*n* = 6) subgroups, such that there were no differences in numbers of aggressive behaviors between the subgroups according to the results of the R–I tests. Subsequently, DW (10 mL/kg), YKS (1.0 g/10 mL/kg), or YKSCH (1.4 g/10 mL/kg) was orally administered daily to rats of each subgroup for 7 days (days 8–14 after Glu injection). S-PB (1 μL) was injected into the right NBM of control rats, and DW (10 mL/kg) was orally administered to the rats once a day for 7 days from days 8–14 after S-PB injection. Aggressive behavior was monitored in all rat groups using the R–I test on day 15 after Glu or S-PB injections.

#### Administration of WAY-100635

In the experiments with WAY-100635, Glu-injected rats (*n* = 36) were divided into three groups on day 8 after injection, such that the total number of aggressive behaviors exhibited by the rats in each group during the R–I test was the same for each group. The rats in these three groups were orally administered either DW (10 mL/kg), YKS (1.0 g/10 mL/kg), or YKSCH (1.4 g/10 mL/kg), and each group was then further divided into two subgroups to receive i.p. injections of saline (vehicle, 1 mL/kg) or WAY-100635 in saline (0.1 mg/1 mL/kg). As a result, rats of all six groups (DW + vehicle, *n* = 6; DW + WAY, *n* = 6; YKS + vehicle, *n* = 6; YKS + WAY, *n* = 6; YKSCH + vehicle, *n* = 6; YKSCH + WAY, *n* = 6) received daily treatments for 7 days (days 8–14 after Glu injections). As controls for the WAY-100635 treatment, a separate group of S-PB-injected rats (*n* = 14) received injections of saline (vehicle, 1 mL/kg, i.p.). Concurrently, DW was administered (10 mL/kg, p.o.) during the schedule for Glu-injected rats. The aggressive behavior was recorded for all animals using R–I tests on day 15 after Glu or S-PB injections.

### Locomotor Activity

On day 8 after Glu injections into the right NBM, rats (*n* = 15) were divided into DW (*n* = 5), YKS (*n* = 5), and YKSCH (*n* = 5) groups with equal numbers of aggressive behaviors. Rats were then orally administered DW (10 mL/kg), YKS (1.0 g/10 mL/kg), or YKSCH (1.4 g/10 mL/kg) once daily on days 8–14 after Glu injection. S-PB-injected rats (*n* = 5) received DW (10 mL/kg) during the same period. Thereafter, the locomotor activities of DW-, YKS-, and YKSCH-administered rats were measured for 24 h on days 12–14 in the home cages using an automated activity counter (NS-AS01; Neuroscience, Tokyo, Japan) placed 15 cm above the cage lid.

### Immunohistochemical Staining for Choline Acetyltransferase (ChAT)

The rats from the locomotor activity experiments were sacrificed for immunohistochemical analyses on day 15 after Glu or S-PB injections. Glu-induced cholinergic neuronal degeneration in the NBM was evaluated using immunohistochemistry according to a previously described procedure (Burk and Sarter, 2001). Briefly, Glu- or S-PB-injected rats were anesthetized with sodium pentobarbital (50 mg/kg, i.p.) and were then transcardially perfused with 100 mL of saline. Subsequent perfusions were performed with 150 mL of 4% paraformaldehyde in 0.1 M phosphate buffer (pH 7.4). Thereafter, the brains were removed, immersed in 30% sucrose in 0.1 M phosphate buffer at 4°C for 72 h, and then frozen at -80°C. Serial coronal sections (30 μm thick) including bilateral NBM areas (1.14–1.41 mm posterior from the bregma) were prepared using a cryostat (Leica Microsystems, Wetzlar, Germany). Choline acetyltransferase (ChAT) was stained as a marker of cholinergic neurons using the conventional flotation method with rabbit anti-ChAT polyclonal antibody (dilution ratio, 1:1000; AB143, Chemicon International, Temecula, CA, USA), biotinylated goat anti-rabbit secondary antibody (PK-6101, Vectastain Elite ABC kit, Vector Laboratories Inc., Burlingame, CA, USA), and avidin-biotin-peroxidase standard complex. Peroxidase activities were then visualized by treating sections with diaminobenzidine tetrahydrochloride dihydrate solution containing hydrogen peroxide and Ni^2+^ (SK-4100, Peroxidase Substrate Kit, Vector Laboratories Inc.). The ChAT-positive cells in the NBM areas (1.0 mm^2^) of each section were microscopically counted by an observer who was blinded to the treatments, and the mean numbers of cells in three serial sections were calculated for each individual NBM area.

### Statistical Analysis

All values are represented as the mean ± SEM. Pairwise comparisons were performed using Student’s *t*-test and multiple comparisons were conducted using one-way ANOVA followed by the *post hoc* Bonferroni test. Differences were considered significant when *p* < 0.05.

## Results

### Development of Aggressive Behaviors Following Glu Injection

**FIGURE 2:**
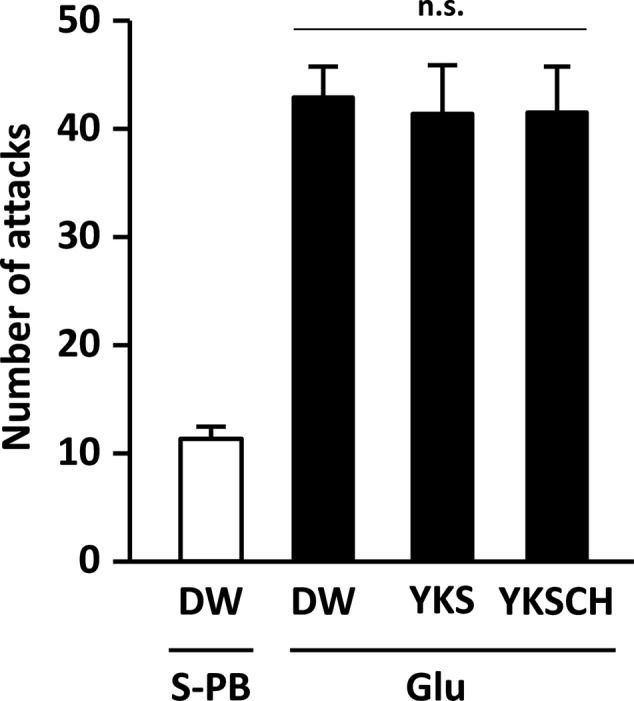
**Development of aggressive behaviors in the rats due to Glu injections into the right side of the NBM.** The number of attacks for Glu-injected rats (*n* = 18) on day 8 after injection was significantly greater than that for S-PB-injected rats (*n* = 8, *p* < 0.001, data not shown). Then, Glu-injected rats exhibiting aggressive behavior were divided into three groups for drug-treatment studies. Data are presented as mean ± SEM; n.s., not significant; S-PB + DW (*n* = 8), Glu + DW (*n* = 6), Glu + YKS (*n* = 6), Glu + YKSCH (*n* = 6).

On day 8 after injection, the number of aggressive behaviors observed for the rats (*n* = 18) that had received the Glu injection into the right NBM was significantly greater than that observed for rats (*n* = 6) that had received the S-PB (vehicle) injection (*p* < 0.001, data not shown). Then, the rats that exhibited aggressive behavior were divided into three groups that did not show a significant difference in the number of aggressive behaviors (**Figure 2**). In our preliminary experiments, the numbers of ChAT-positive cells in the NBM of rats injected with Glu were significantly lower on day 8 than those for S-PB injected rats, and these observations were reflected in subsequent experiments on day 15 (see later, **Figures 6, 7**).

### Effects of YKS and YKSCH on Glu-induced Aggressive Behaviors

**FIGURE 3:**
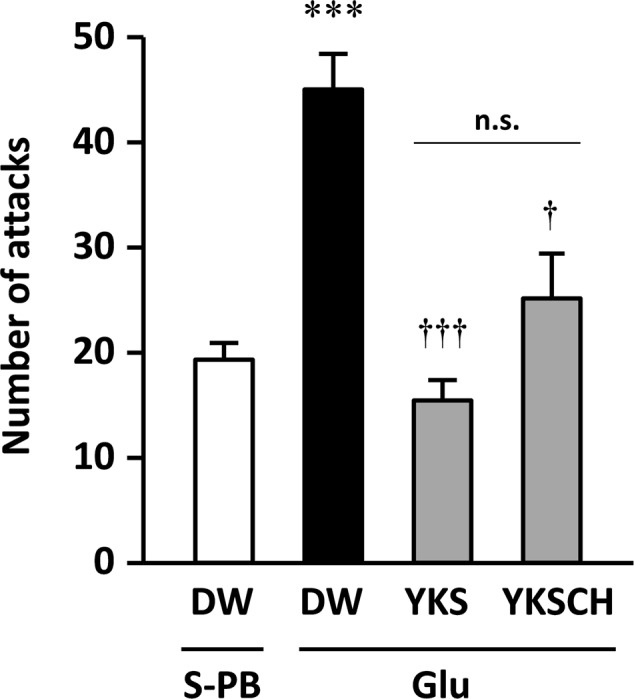
**Therapeutic effects of YKS and YKSCH on the aggressive behavior of Glu-injected rats.** Glu-injected rats were treated orally with distilled water (DW), YKS, or YKSCH for 7 days (days 8–14 after Glu injection). S-PB-injected rats received DW during the same period as Glu-injected rats. Aggressive behaviors were evaluated using R–I tests on day 15. Data are presented as mean ± SEM; ^∗∗∗^*p* < 0.001 vs. S-PB + DW, and ^†^*p* < 0.05, ^†††^*p* < 0.001 vs. Glu + DW; n.s., not significant; S-PB + DW (*n* = 8), Glu + DW (*n* = 6), Glu + YKS (*n* = 6), Glu + YKSCH (*n* = 6).

On day 15 of the experiment, the number of aggressive behaviors observed was significantly greater for the Glu-injected rats that were administered DW than for the S-PB-injected rats that were administered DW [*F*(3,22) = 11.500, *p* < 0.001; **Figure 3**]. These data were consistent with those shown in **Figure 2**, and the increased aggressive behaviors were significantly reduced following seven daily treatments with YKS or YKSCH [*F*(3,22) = 11.500, *p* < 0.001, and *p* < 0.05, respectively]. The numbers of aggressive behaviors observed did not differ between YKS- and YKSCH-administered rats.

### WAY-100635 Abolishes the Effects of YKS and YKSCH on Aggressive Behaviors

**FIGURE 4:**
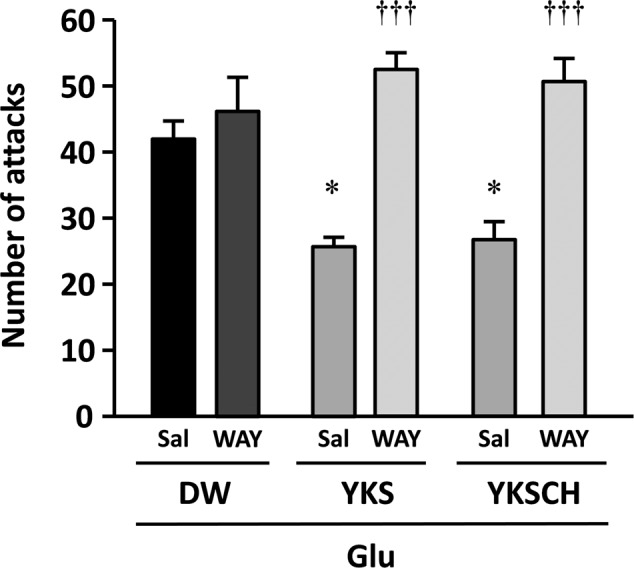
**The 5-HT_1A_ receptor antagonist WAY-100635 abolishes the anti-aggressive effects of YKS and YKSCH.** Glu-injected rats exhibiting aggressive behaviors were divided into three treatment groups (DW, YKS, and YKSCH), and these were further divided into WAY-100635 treatment and saline control groups. Seven days after the Glu or S-PB injections, DW, YKS, or YKSCH was administered orally and saline or WAY-100635 was administered by intraperitoneal (i.p.) injections for 7 days (days 8–14 after Glu or S-PB injections). Data are presented as mean ± SEM (*n* = 6); ^∗^*p* < 0.05 vs. saline in DW group; ^†††^*p* < 0.001 vs. saline in each group; Sal, saline; WAY, WAY-100635.

To investigate the mechanisms behind the effects of YKS and YKSCH on Glu-induced aggressive behaviors, we coadministered the 5-HT_1A_ receptor antagonist WAY-100635. The numbers of aggressive behaviors observed did not differ between S-PB-injected rats administered WAY-100635 (17 ± 3 counts/10 min) and those administered saline (vehicle; 19 ± 2 counts/10 min). Moreover, the numbers of aggressive behaviors did not differ between Glu-injected rats that received (p.o.) DW with i.p. injections of saline or WAY-100635 (**Figure 4**). However, the number of aggressive behaviors observed was significantly lower in Glu-injected rats that had received YKS and saline compared with those that had received DW and saline [*F*(5,30) = 13.720, *p* < 0.05]. This ameliorative effect of YKS was significantly counteracted by coadministration of WAY-100635 [*F*(5,30) = 13.720, *p* < 0.001]. Moreover, for the rats that had received YKSCH and saline, the number of aggressive behaviors was significantly lower than for those administered DW and saline [*F*(5,30) = 13.720, *p* < 0.05]. This ameliorative effect of YKSCH was also significantly counteracted by coadministration of WAY-100635 [*F*(5,30) = 13.720, *p* < 0.001].

### Effects of YKS and YKSCH on Locomotor Activities of Glu-Injected Rats

**FIGURE 5:**
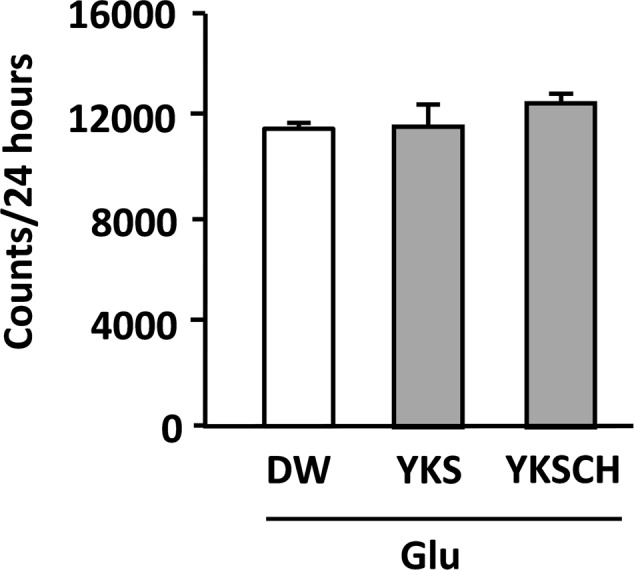
**Effects of YKS and YKSCH on locomotor activities of Glu-injected rats.** Locomotor activities of drug-administered rats following injections of Glu into the NBM were measured for 24 h periods on days 12–14. Data are presented as mean ± SEM (*n* = 5).

The locomotor activities of Glu-injected rats following administration of DW, YKS, or YKSCH were measured in the home cages on days 12–14 after Glu injection. The total activities per 24 h did not differ significantly between experimental groups (**Figure 5**), and were similar during light-on and light-off periods (data not shown).

### Effects of YKS and YKSCH on Numbers of ChAT-Positive Cells in NBM Areas

**FIGURE 6:**
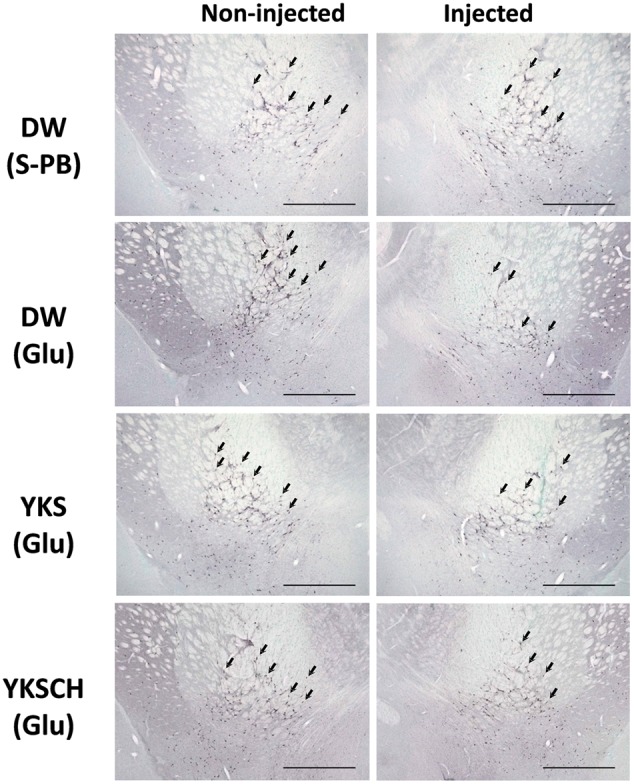
**Representative microphotographs of ChAT-positive cells in the NBM of control and Glu-injected rats following treatment with DW, YKS, or YKSCH.** Control rats were injected with S-PB. DW, YKS, or YKSCH was administered to rats for 7 days from days 8–14 after Glu or S-PB injections. Rats were sacrificed on day 15, and ChAT immunostaining analyses were performed on the excised brains. Arrows indicate ChAT-positive cells in the NBM. Scale bars indicate 1.0 mm.

The immunoreactivities of ChAT were examined in the NBM areas of the brains of drug-administered rats on day 15 after the Glu or S-PB injection into the right NBM. Representative microphotographs of cells stained for ChAT from the NBM of control rats (injected with S-PB and administered DW) exhibit no differences between the numbers of ChAT-positive cells in the injected (right) and non-injected (left) sides of the NBM (**Figure 6**). In contrast, among the rats that had been injected with Glu and administered DW, the numbers of ChAT-positive cells in the injected side were fewer than in the non-injected side. Moreover, similar reductions in ChAT-positive cell numbers were observed in YKS- and YKSCH-administered rats.

**FIGURE 7:**
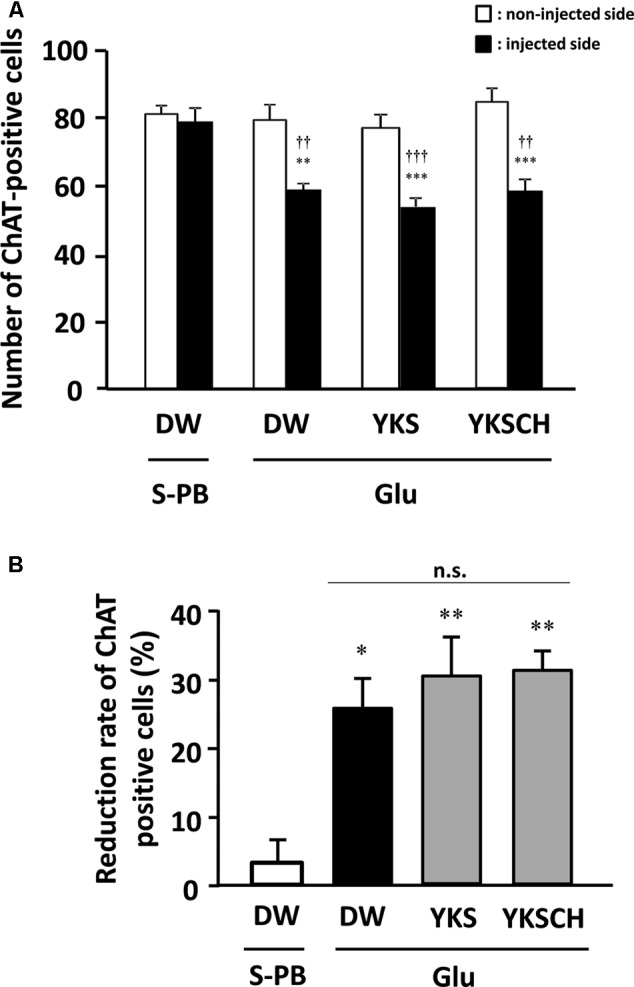
**(A)** Effects of YKS and YKSCH on the numbers of ChAT-positive cells in the injected and non-injected sides of the NBM. ChAT-positive cells were counted in 1.0 mm^2^ areas from injected (black columns) and non-injected (white columns) sides of the NBM. Data are presented as mean ± SEM (*n* = 5); ^∗∗^*p* < 0.01, ^∗∗∗^*p* < 0.001 vs. non-injected sides of the NBM, and ^††^*p* < 0.01, ^†††^*p* < 0.001 vs. injected sides of the NBM in control (S-PB-injected and DW-administered) rats. **(B)** Reduction rates of ChAT-positive cells for the injected sides of the NBM were calculated relative to the numbers of ChAT-positive cells in non-injected sides of the NBM. Data are presented as mean ± SEM (*n* = 5 for each group); ^∗^*p* < 0.05, ^∗∗^*p* < 0.01 vs. S-PB + DW, n.s., not significant.

In control rats, the numbers of ChAT-positive cells in the S-PB-injected sides of the NBM were not significantly different from those in the non-injected sides (**Figure 7A**). However, in DW-administered rats, the numbers of ChAT-positive cells in the Glu-injected sides of the NBM were significantly lower than those in the non-injected sides [*F*(7,32) = 12.340, *p* < 0.01]. Moreover, YKS- or YKSCH-administered rats had significantly fewer ChAT-positive cells in the injected sides than in the non-injected sides [*F*(7,32) = 12.340, *p* < 0.001; *F*(7,32) = 12.340, *p* < 0.001, respectively]. In addition, the numbers of ChAT-positive cells in the injected sides of the NBM were significantly lower in the DW-, YKS-, and YKSCH-administered rats than in the control rats [*F*(7,32) = 12.340, *p* < 0.01, *p* < 0.001, *p* < 0.01, respectively], although no significant differences were identified between the three treatment groups.

As shown in **Figure 7A**, the numbers of ChAT-positive cells in the injected NBM of DW-, YKS-, and YKSCH-administered rats were significantly lower than in the non-injected NBM. In subsequent analyses, ChAT-positive cell reduction rates (**Figure 7B**) for the injected NBM were calculated relative to the non-injected NBM. These data showed significantly greater reduction rates of ChAT-positive cells in the injected sides of the NBM of DW-, YKS-, or YKSCH-administered rats compared with those in the control (S-PB-injected and DW-administered) rats [*F*(3,16) = 9.452, *p* < 0.05, *p* < 0.01, *p* < 0.01, respectively]. In addition, the reduction rates did not differ significantly between these treatment groups.

## Discussion

In the present study, we provided three new findings. Firstly, cholinergic degeneration in the unilateral NBM induced by intracranial injection of Glu could develop aggressiveness in the rats. Secondly, the Glu-induced aggressiveness was ameliorated by the treatment of YKS or YKSCH, in which the therapeutic effects of YKSCH were the almost same as that of YKS. Finally, the therapeutic effects of YKS and YKSCH were inhibited by the treatment of the 5-HT_1A_ receptor antagonist, suggesting the underlying mechanisms through 5-HT_1A_ receptor stimulation.

The NBM is an area of the substantia innominata of the basal forebrain, from which resident cholinergic neurons are widely projected into the neocortex, which contains large amounts of ACh and its synthetic enzyme ChAT (Mesulam et al., 1983). In AD patients, cholinergic dysfunction in the frontal and temporal cortices has been correlated with BPSD such as aggressiveness (Garcia-Alloza et al., 2005), and glutamate excitotoxicity is suggested to be involved in the cholinergic degeneration (Maragas et al., 1987; Arendt et al., 1995; Francis, 2003; Montiel et al., 2005; Bohnen and Albin, 2011). In the present study, we found that aggressive behaviors had developed by day 8 and persisted until day 15 after Glu injection (**Figures 2, 3**), and the similar numbers of aggressive attacks on these days suggest that Glu-induced aggressiveness did not progress after day 8. On day 15, we also found 25–31% decreases in the numbers of cholinergic neurons at the injected sides of the NBM compared with the non-injected sides (**Figures 6, 7**). These results suggest that partial cholinergic lesions in the NBM are involved in the development of aggressive behaviors. In our preliminary studies, the aggressive behaviors of rats that had received Glu injections in both sides of the NBM were similar to those of rats that had received injections at only the right side of the NBM. Furthermore, anxiety and hypoactivity were reported to develop after unilateral injections of *N*-methyl-D-aspartate or β-amyloid peptide into the NBM (Harkany et al., 2000, 2001). These data suggest that aggressiveness in the present rats was not triggered by bilateral lesional imbalances between the injected and non-injected sides. In addition, the aggressive behaviors of Glu-injected rats that were treated with DW were not abolished by administration of WAY-100635 (**Figure 4**), indicating that 5-HT_1A_ receptors are not involved in the aggressiveness of rats with cholinergic lesions. In previous studies using mice models, aggressive behaviors that were induced by isolation and aversive stimuli (tail pinching) were suppressed by the muscarinic ACh receptor antagonist scopolamine (DaVanzo et al., 1966). These studies suggest that hyperfunction of cholinergic activity may contribute to the development of aggressive behaviors. Considering that the rats having cholinergic degeneration in the NBM showed aggressive behaviors in the present study, the decrease or increase in cholinergic activity might induce aggressiveness. Nonetheless, it should be noted that our rat model is useful for elucidation of the BPSD pathophysiology and evaluation of psychopharmacological efficacy of drugs because cholinergic degeneration in the NBM is well-known as a pathogenic factor of AD.

Treatment of the aggressive rats with YKS or YKSCH for 7 days significantly ameliorated their aggressive behavior (**Figure 3**). However, these effects did not reflect inhibition of general physical activity, because both treatment groups had normal locomotor activity on days 12–14 after Glu injection (**Figure 5**). Previous studies have demonstrated that YKS protects against aggressive behaviors in several animal models of neurological disorders (Ikarashi et al., 2009; Takeda et al., 2008, 2012; Iizuka et al., 2010; Fujiwara et al., 2011; Sekiguchi et al., 2011; Nishi et al., 2012). In agreement with these reports, YKS and YKSCH had therapeutic effects on aggressiveness in the present animal model of AD. YKSCH is traditionally used for the treatment of neurotic patients with greater concurrent impairments of physical strength and more chronic conditions than those for which YKS is administered. However, the present effects of YKSCH were similar to those of YKS, suggesting that the anti-aggressive actions of YKSCH are due to the effects of YKS. These data suggest that further comparisons of these drugs in other animal models of aggression should be performed.

The effects of YKS and YKSCH on aggressive behavior were abolished following coadministration of the 5-HT_1A_ receptor antagonist WAY-100635, suggesting that YKS and YKSCH stimulate the 5-HT_1A_ receptor. We previously reported that geissoschizine methyl ether (GM), which is an indole alkaloid derived from the *Uncaria* hook constituent of YKS and YKSCH, has partial agonistic effects on 5-HT_1A_ receptors (Nishi et al., 2012). Moreover, GM- and YKS-mediated protection against aggressive behaviors in isolation-stressed mice was counteracted by WAY-100635, suggesting that these anti-aggressive effects are mediated by 5-HT_1A_ receptor stimulation (Nishi et al., 2012). GM crosses the blood–brain barrier after oral administration of YKS (Imamura et al., 2011; Kushida et al., 2013), and *in vitro* autoradiographic analysis using tritium-labeled GM revealed that GM binds to 5-HT_1A_ receptors in rat frontal cortical regions and the dorsal raphe nucleus (Mizoguchi et al., 2014). Because 5-HT_1A_ receptor stimulation increases ACh release in the prefrontal cortex (Katsu, 2001; Millan et al., 2004), a cholinergic regulatory mechanism of GM that operates through 5-HT_1A_ receptor stimulation in the frontal cortex may be associated with the present therapeutic actions of YKS and YKSCH. However, YKS and YKSCH did not restore the number of cholinergic neurons in the Glu-injected NBM of rats (**Figures 6, 7**), indicating that the therapeutic effects of YKS and YKSCH on aggressiveness are not due to recovery from glutamate-mediated cholinergic neuronal degeneration.

## Conclusion

Herein, we have shown that Glu injection into the NBM leads to the development of aggressive behaviors and cholinergic degeneration in rats, and that YKS and YKSCH have similar therapeutic effects on aggressiveness. Subsequent experiments suggested that these effects are mediated by 5-HT_1A_ receptor stimulation.

## Author Contributions

All authors listed, have made substantial, direct and intellectual contribution to the work, and approved it for publication.

## Conflict of Interest Statement

The authors declare that the research was conducted in the absence of any commercial or financial relationships that could be construed as a potential conflict of interest.
